# Inadvertent steroid injection into the crystalline lens

**DOI:** 10.3205/oc000067

**Published:** 2017-06-27

**Authors:** Md. Shahid Alam, Vikas Khetan

**Affiliations:** 1 Orbit, Oculoplasty, Reconstructive & Aesthetic Services, Medical Research Foundation, Sankara Nethralaya, Chennai, India; 2 Bhagwan Mahavir Vitreoretina Services, Medical Research Foundation, Sankara Nethralaya, Chennai, India

**Keywords:** intralenticular, steroid, triamcinolone

## Abstract

Intravitreal triamcinolone is administered for a wide number of vitreoretinal conditions. Several complications including cataract formation, raised intraocular pressure, and endophthalmitis have been reported following intravitreal injections. We report a rare case wherein triamcinolone was inadvertently injected directly into the crystalline lens. A 41-year-old male presented to us with a history of intravitreal injection of triamcinolone in the left eye 2 weeks earlier. Slit lamp examination revealed a needle tract in the crystalline lens with steroid granules dispersed throughout the lens core. Such a complication is extremely rare with only three cases reported previously.

## Introduction

Intravitreal triamcinolone injections are administered in the treatment of an increasing number of vitreoretinal conditions namely diabetic macular edema, cystoid macular edema following vein occlusions, and retinal vasculitis [1], [2], [3]. Several complications including cataract formation, raised intraocular pressure, and endophthalmitis have been reported following intravitreal injection of triamcinolone [4], [5], [6]. Inadvertent injection of the drug directly into the crystalline lens is a very rare complication and to the authors’ best knowledge has only been reported three times in the literature [7], [8], [9]. We herewith report the fourth case.

## Case description

**Figure 1:**
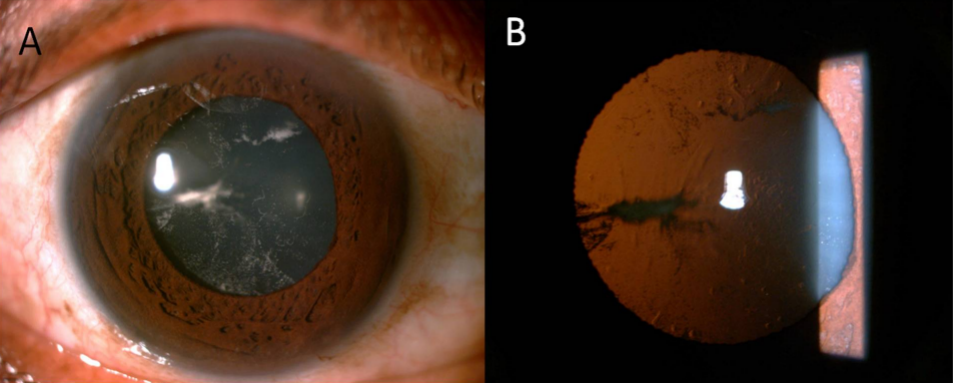
A: Multiple linear intralenticular opacities consistent with intralenticular steroid along with needle tract at 9 o’clock position. B: The needle tract seen more clearly on retroillumination.

A 41-year-old male presented to us with a history of receiving intravitreal triamcinolone in the left eye 2 weeks earlier. The patient was a known case of retinal vasculitis and had received oral steroids and intravitreal bevacizumab in the past. On examination, the best corrected visual acuity in the left eye was 20/200. Slit lamp examination revealed multiple linear lenticular opacities with a needle tract at 9 o’clock, consistent with intralenticular steroid within the lens core (Figure 1 (Fig. 1)). There was no cataract formation. The anterior segment was otherwise quiet in both eyes and the intraocular pressure was within normal limits.

Indirect ophthalmoscopy revealed a macular edema with multiple dot blot hemorrhages and laser marks in the left eye. The right eye was unremarkable except for some old healed vasculitis marks. 

The patient was planned for intravitreal bevacizumab injection in the left eye after a complete systemic workup for vasculitis. The patient was informed about the possibility of cataract formation in the future. He was then lost to follow-up.

## Discussion

The reported incidence of iatrogenic lenticular injury following intravitreal injection ranges from 0–0.07% [10], [11], [12]. Though the reported incidence for cataract formation and lenticular touch after intravitreal injection is quite low, inadvertent injection directly into the crystalline lens is even rarer and only three such instances have been reported in the literature till date.

Rajak SN et al. reported a case of an 82-year-old phakic male who was planned for intravitreal triamcinolone for persistent chronic diabetic macular edema [7]. The injection was given 3 mm posterior to the superotemporal limbus. Routine examination 1 hour post procedure revealed a needle puncture site in the superotemporal posterior capsule with multiple collections of triamcinolone throughout one lamellar plane of posterior lens substance. Fortunately, the cataract did not progress until approximately one year of follow-up and the patient did not require a cataract surgery.

Jalil A et al. reported a case of an 87-year-old male suffering from macular subretinal neovascular membrane secondary to age-related macular degeneration [8]. Intravitreal triamcinolone injection was given 4 mm posterior to the limbus in the inferotemporal quadrant. During the injection. the surgeon noted a white wave throughout the lens, similar to a hydrodissection wave of phacoemulsification, suggestive of intralenticular triamcinolone injection. The patient later underwent a successful phacoemulsification with intraocular lens implantation.

The third case of inadvertent intralenticular steroid injection was reported by Koller S et al. wherein Ozurdex implant was inadvertently injected into the crystalline lens of a 63-year-old woman suffering from branch retinal vein occlusion and macular edema [9]. The patient later underwent successful phacoemulsification with intraocular lens implantation for cataract developed from lenticular trauma.

Iatrogenic lenticular puncture usually causes a rapid cataract formation, sometimes along the tract of perforation. In our case and the case reported by Rajak SN et al. [7], the injection site as well as the tract along which the triamcinolone granules travelled were very clearly visible. We propose that, since the lenticular capsule was intact and the drug was injected directly into the lens core without capsular rupture, there was no cataract formation even after two weeks of lenticular injury.

To minimize the possible risk of lenticular injuries and inadvertent intralenticular injections, one has to pay close attention to three crucial components of intravitreal injection. They include a 4 mm distance from the limbus, trajectory of the needle directed towards the vitreous cavity, and visualization of the needle tip within the pupil [7]. Since the crystalline lens is not exposed to aqueous or blood circulation, hence there is no chance of sequestrated steroid being absorbed, and the patient finally needs a cataract extraction in the majority of cases.

## Notes

### Competing interests

The authors declare that they have no competing interests.
